# A surgical case of anti-coagulant ileus mimicking small-bowel tumors: a case report

**DOI:** 10.1186/s40792-023-01795-5

**Published:** 2024-01-02

**Authors:** Hideyuki Masui, Hiroyuki Kobayashi, Yusuke Sakamoto, Shintaro Yagi, Satoshi Kaihara

**Affiliations:** 1 Department of Surgery, Kobe City Medical Center General Hospital, 2-1-1Minatojima-Minamimachi, Chuo-Ku, Kobe, 650-0047 Japan; 2 Department of Gastrointestinal Surgery, Graduate School of Medicine, Kyoto University, 54 Shogoin-Kawahara-Cho Sakyo-Ku, Kyoto, 606-8507 Japan; 3 Department of Hepato-Biliary-Pancreatic and Transplantation, Kanazawa University, 13-1 Takaramachi, Kanazawa City, Ishikawa 920-0965 Japan

**Keywords:** Anti-coagulant ileus, Anti-coagulant therapy, Over-anticoagulation, Warfarin, Spontaneous intramural small-bowel hematoma

## Abstract

**Background:**

Anti-coagulant ileus, characterized by intramural hematoma due to excessive anti-coagulant therapy, presents a diagnostic challenge. Although previously considered uncommon, recently, reporting cases of anti-coagulant ileus have become more frequent. Herein, we report a rare surgical case of anti-coagulant ileus mimicking small-bowel tumors.

**Case presentation:**

A 79-year-old man was admitted to our hospital for fatigue. He had been administered warfarin for 5 months for atrial fibrillation. On admission, the patient exhibited mild epigastric tenderness. Laboratory test results revealed anemia (hemoglobin, 8.4 g/dL); unmeasurably prolonged prothrombin time (PT) with international normalized ratio (INR) > 8; and elevated soluble interleukin 2 receptor (sIL-2R) levels (849 IU/mL; normal range, 122–496 IU/mL). Abdominal plain computed tomography (CT) showed a circumferentially thickened intestinal wall at one site in the jejunum and two in the ileum. After hospitalization, bowel obstruction did not improve with conservative treatment. Suspecting small-bowel tumors such as lymphoma, the patient subsequently underwent open surgery on day 3 after admission. No obvious tumor mass was observed intra-operatively. However, only thickened and hemorrhagic segments were identified at the suspected sites. We performed partial jejunal and ileal resections of 12 and 27 cm, respectively. Histopathology confirmed submucosal congestion, edema, and hemorrhage in each area without tumor components, leading to the final diagnosis of intramural hematoma. The postoperative course was uneventful, and he was discharged on postoperative day 9. No recurrence occurred during the 5-year follow-up period.

**Conclusions:**

We encountered a surgical case of anti-coagulant ileus, which was difficult to differentiate from malignant lymphoma based on CT findings and high sIL-2R levels. The possibility of anti-coagulant ileus should always be considered in patients on long-term anticoagulation medication and bowel obstruction with high PT-INR values.

## Background

Anti-coagulant ileus, which is ileus caused by an intramural hematoma due to excessive anti-coagulant therapy, was first described by Hafner et al. in 1962 [1]. On the other hand, intramural hematoma of the small intestine was identified as a complication of blunt trauma, particularly in children. This nontraumatic type of intramural intestinal hematoma, previously thought to be an uncommon complication of anti-coagulant treatment, has now been more frequently reported because of the accessibility of modern image-based diagnostic tools and increases in number of patients receiving anti-coagulant medication. Herein, we present a rare surgical case of anti-coagulant ileus mimicking small-bowel tumors.

## Case presentation

**Fig. 1:**
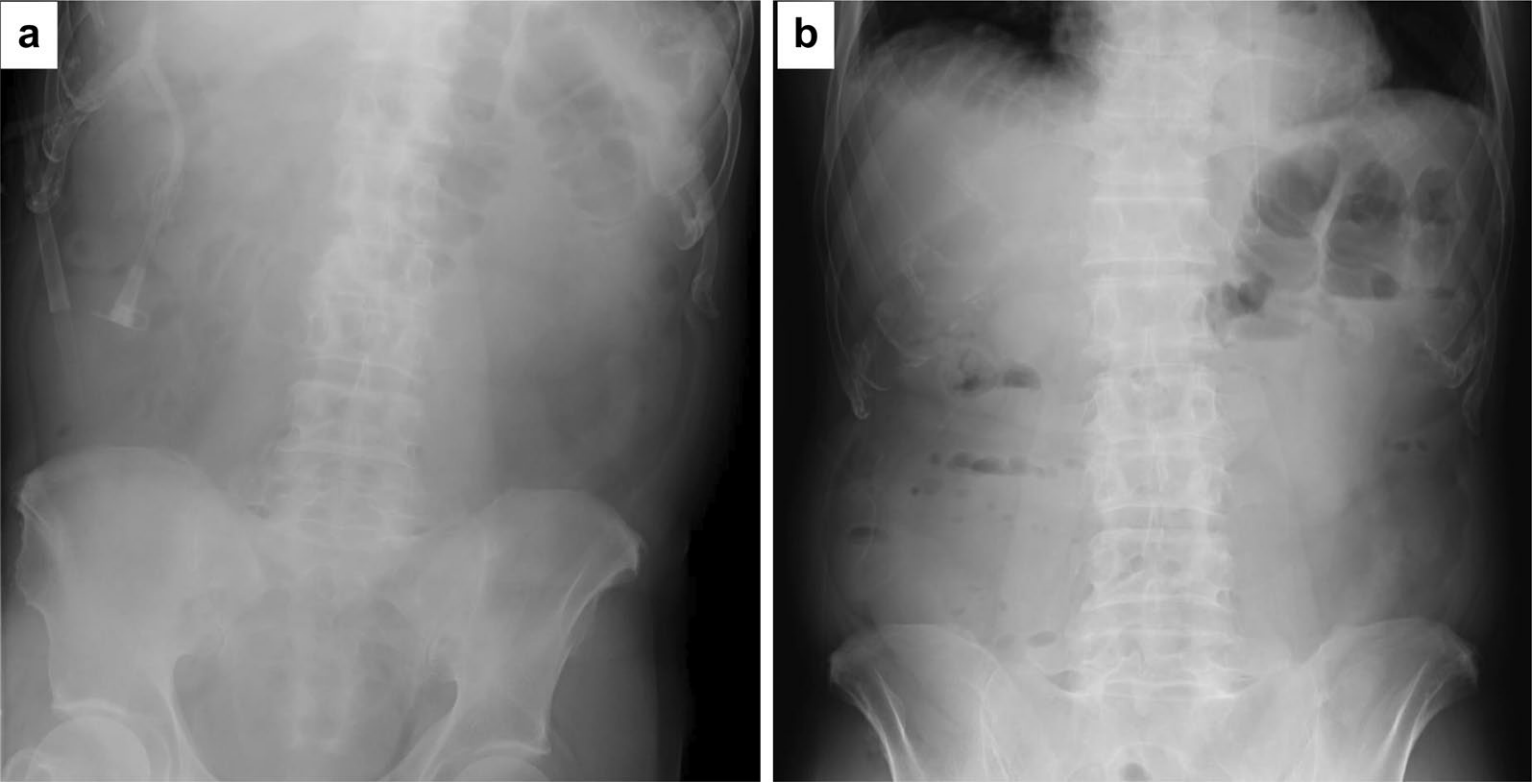
Plain X-ray of the abdomen. Plain X-ray shows a dilated intestine with a step ladder sign, suggesting small-bowel obstruction (**a**). In the standing position, niveau formation can be clearly recognized (**b**)

**Fig. 2:**
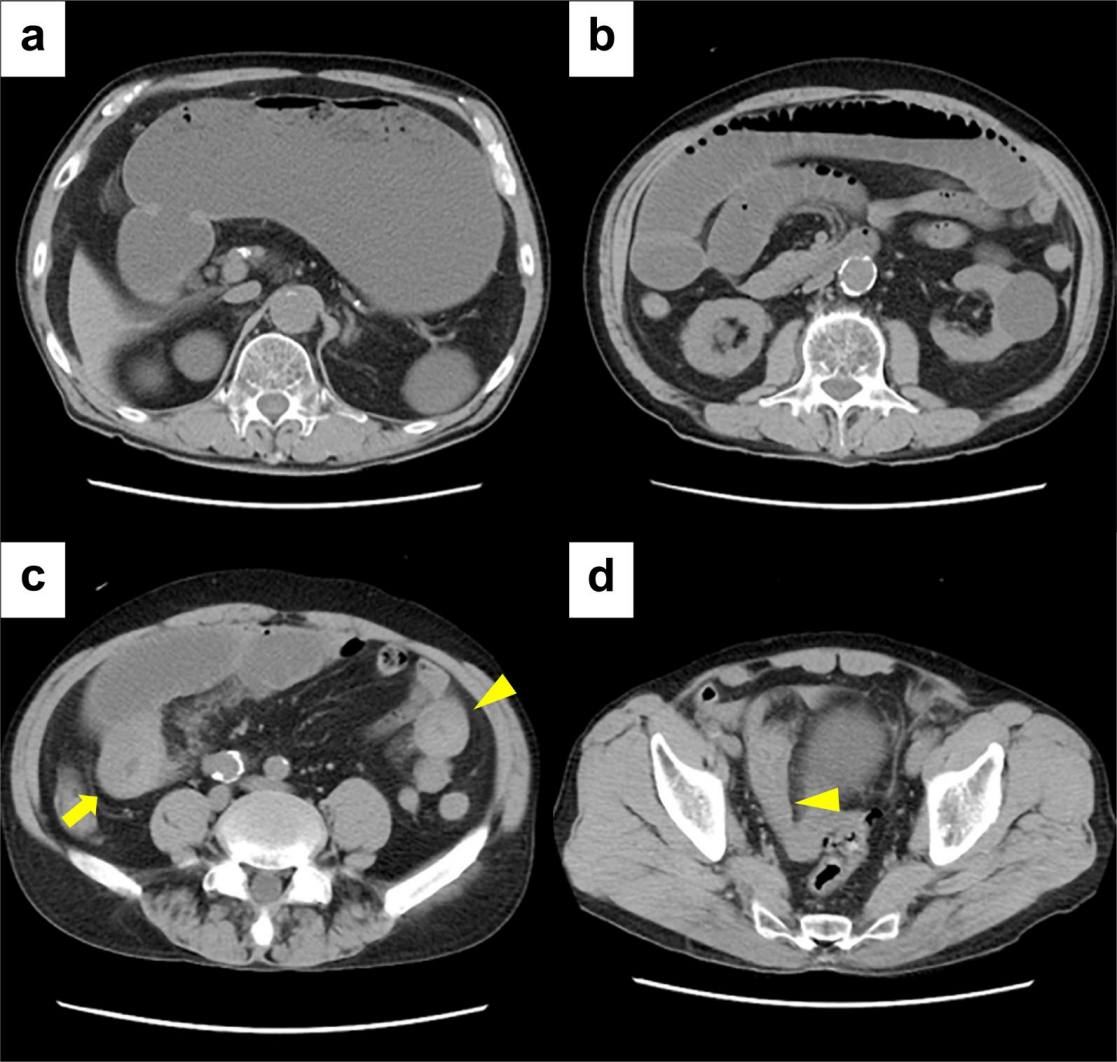
Plain CT scanning images. Plain CT demonstrates notable gastric dilatation (**a**) and extensive small-bowel dilatation (**b**). In addition, the images display circumferentially thickened wall at the jejunum (arrow) and the ileum (arrowhead) (**c**, **d**), suggesting these to be the underlying causes of the intestinal obstruction

The patient was a 79-year-old man with a history of atrial fibrillation, hypertension, congestive heart failure, type 2 diabetes mellitus, and chronic kidney disease. The patient had been administered warfarin (2.5 mg/day) regularly for 5 months, wherein the International Normalized Ratio (INR) value was 1.57 on the most recent follow-up visit. He had no history of surgery, trauma, coughing, or vigorous activity. He was admitted to our hospital for fatigue that began 4 days earlier. The following vital signs were unremarkable: blood pressure, 136/90 mmHg; pulse rate, 107 beats/min; and oxygen saturation, 96% (on room air). On physical examination, he exhibited only slight epigastric discomfort without signs of peritoneal irritation. Laboratory blood analysis revealed mild anemia (hemoglobin [Hb], 8.4 g/dL); elevated white cell count (white blood cell, 17,700 cells/mL); and C-reactive protein (CRP, 6.25 mg/dL). Worsened renal function (blood urea nitrogen: 58.2 mg/dL and creatinine: 3.42 mg/dL) and unmeasurably prolonged prothrombin time (PT) with INR > 8 were also found. An X-ray film of the abdomen revealed intestinal dilation with a large amount of gas, indicating obstruction of the small intestine (Fig. 1). Abdominal plain computed tomography (CT) showed a circumferentially thickened intestinal wall at one site in the jejunum and two in the ileum (Fig. 2). However, contrast-enhanced CT could not be performed because of previously recognized renal insufficiency. The possibility of a small intestinal tumor, such as lymphoma causing bowel obstruction, was considered as the soluble interleukin 2 receptor (sIL-2R) level was elevated at 849 IU/mL (normal range 122–496 IU/mL).

**Fig. 3:**
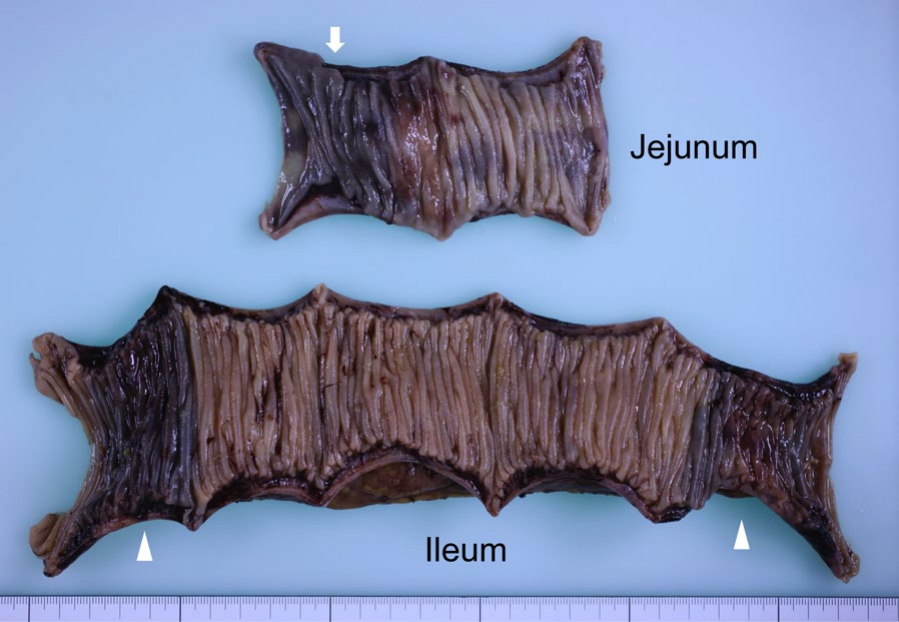
Gross image of the resected specimen. The resected specimen includes two parts; the upper shows brown-colored lesions in the jejunum (one site), indicative of submucosal congestion, edema, and hemorrhage, while the lower part reveals similar findings in the ileum (two sites)

**Fig. 4:**
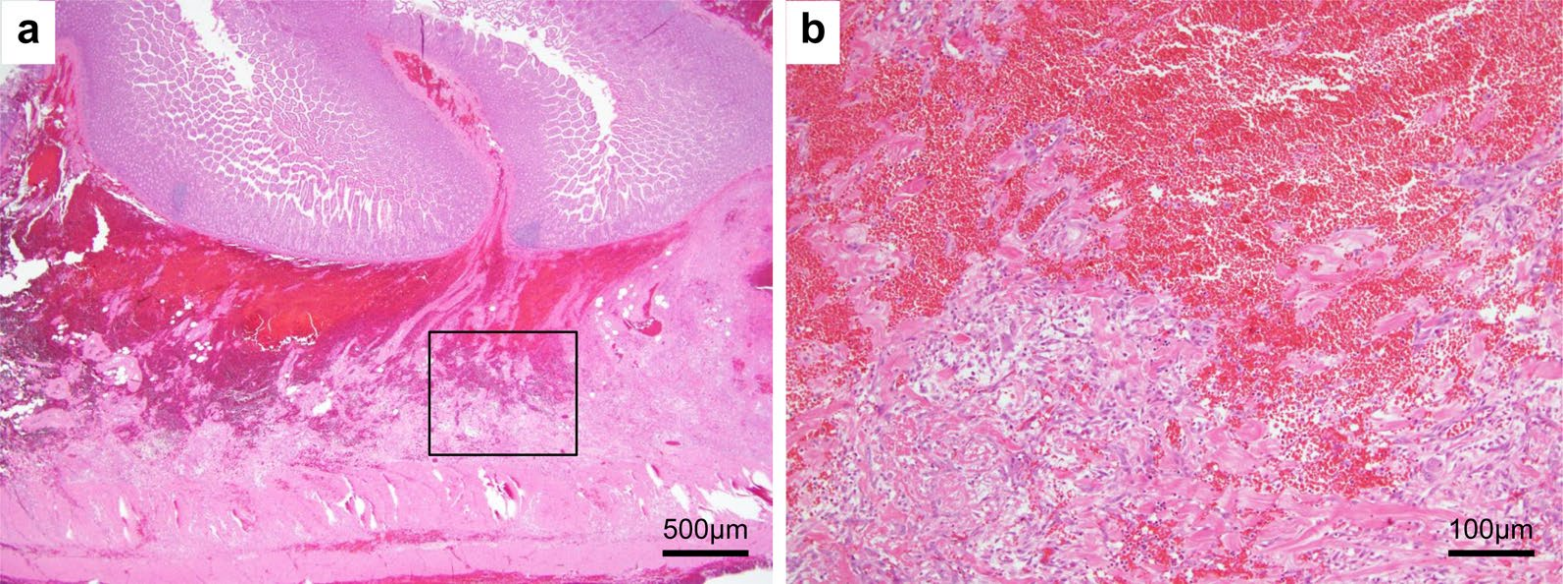
Histopathological observations. Histopathological findings of the specimen reveals a hematoma mainly localized in the submucosal layer (**a**), establishing the diagnosis of anti-coagulant ileus. Strongly magnified images show no tumor components (**b**)

On the first day after admission, oral feeding was ceased and decompression using an ileus tube was performed. Warfarin was discontinued. In addition, fresh frozen plasma (FFP) and vitamin K were administered intravenously. His anemia showed signs of recovery (Hb 10.2 g/dL) without blood transfusion. The PT-INR value was also corrected to 2.18. However, no improvement in the ileus was observed after 2 days, as indicated by the lack of reduction in gastric tube drainage. Under the suspicion of small-bowel tumors such as lymphoma, the patient subsequently underwent semi-emergency open surgery to remove the lesions on day 3 after admission. The intra-operative findings revealed minimal bloody ascites in the peritoneal cavity without an apparent mass suggestive of a tumor. Only thickened and hemorrhagic segments of the jejunum and ileum were observed at the suspected sites. Finally, we performed partial jejunal and ileal resections of 12 and 27 cm, respectively. Moreover, we performed functional end-to-end anastomosis at two sites with drainage of the intra-abdominal blood. The resected specimen showed brown thickened lesions with occlusion of the lumen (Fig. 3). Histopathology confirmed submucosal congestion, edema, and hemorrhage on hematoxylin and eosin staining (Fig. 4). The final diagnosis was small intestinal intramural hematoma, without tumor components.

The postoperative course was uneventful: the patient began oral feeding and resumed the same amount of warfarin as before the surgery on postoperative day (POD) 4. The patient was discharged on POD 9, wherein no recurrence occurred during the 5-year follow-up period.

## Discussion

Anti-coagulant ileus, characterized by spontaneous intramural small-bowel hematoma (SISBH), is induced by the over-anticoagulation of warfarin, while such a hematoma is rarely caused by antiplatelet agents or heparin [2]. In addition, Non-Vitamin K Oral Anticoagulants (NOACs) such as apixaban, dabigatran, rivaroxaban, and edoxaban have become widely accepted and are commonly used, all of which usually do not require laboratory monitoring. Of note, the first three NOACs have been found to be linked to gastrointestinal hematomas [3–5].

Although the jejunum and ileum are the most commonly involved [2], cases of intramural hematoma in the esophagus and colorectum have also been reported [6, 7]. These characteristics differ from traumatic small-bowel hematomas, which predominantly affect the duodenum and tend to be focal [8]. The most likely cause is a ruptured terminal artery, which leads to bleeding from the submucosa and dissection of the muscular layers [9]; unlike in mesenteric vascular occlusion, the mucosa usually remains viable [10].

The clinical manifestations are variable: occlusive symptoms or gastrointestinal hemorrhage [11]. The traditional clinical triad of abdominal pain, bowel obstruction, and multiple bleeding signs may not be present in all patients. The most frequent manifestation is bowel obstruction, often incomplete. In addition, the clinical symptoms may differ depending on the location of hematoma. Therefore, establishing an accurate diagnosis poses a challenge due to various clinical manifestations.

Patients with anti-coagulant ileus frequently have an elevated INR [8]. Another common observation is the presence of leukocytosis and anemia. In an analysis of 13 patients, only one (7.6%) had anemia at admission, while 11 (84.6%) developed anemia within the first 48 h after hospitalization due to fluid resuscitation [12].

Imaging modalities, such as CT and ultrasonography (US), are generally utilized to confirm diagnosis. Several radiographic features are described: thickening of the intestinal wall > 1 cm, thickened mucosal folds, and picket-fence appearance with oral contrast [8]. Although the presence of intramural hyperdensity on non-contrast CT is an accurate indicator of hemorrhage, gastrointestinal bleeding was not observed in our case. An enhanced CT shows a doughnut-shaped thickening of the intestinal wall, with a three-layer structure reflecting the muscular layer, submucosa, and mucosa [13]. Nevertheless, in the presented case, the use of contrast agents was not feasible due to impaired renal function, a common issue among warfarin users.

Moreover, the sIL-2R level (normal range, 122–496 IU/mL) was elevated in the case, which suggested the possibility of a lymphoid neoplasm [14]. Furthermore, the image on the unenhanced CT scan resembled that of a malignant small intestinal lymphoma, complicating the preoperative diagnosis. Under the suspicion of a small-bowel malignant lymphoma, an exploratory laparotomy was performed. However, pathological findings unexpectedly confirmed a final diagnosis of anti-coagulant ileus.

The basic therapy for intramural hematoma is conservative management: discontinuation of the anti-coagulant; vitamin K administration with or without FFP; use of proton pump inhibitors; treatment of anemia and electrolyte imbalances; and decompression using nasogastric or ileus tubes [11]. Total parenteral nutrition may be necessary for prolonged starvation. Coagulation markers usually return to the normal range within 72 h with the treatment of 2–4 units of FFP and vitamin K [15]. Vitamin K should be administered with caution because it could potentially trigger a rebound effect, leading to increased hypercoagulability and risk of thrombotic diathesis [8, 10, 12]. In most cases, the symptoms improve within 4–6 days [11]. In addition, hematoma resolves after 3 weeks [8], indicative of a reversible phenomenon. Other underlying mechanisms should be considered if the condition lasts for more than 2 months [16, 17].

Indications for surgery include a malignancy-like appearance; active intramural or intraperitoneal hemorrhage; perforation; severe ischemia; or failure to resolve symptoms within a week [12]. Although our case lacked urgent surgical indications, the persistent ileus led us to a semi-emergent operation. If included in our preoperative differential diagnosis, conservative treatment and further diagnostic tests such as US or Fluorodeoxyglucose Positron Emission Tomography (FDG-PET) might have been considered. It is worth noting that surgery could still have been a viable option following any improvement in ileus and coagulation abnormalities.

The short-term outcomes in patients with SISBH are generally favorable, except for those with sepsis due to associated medical comorbidities [18]. Significant hematomas involving more than half the small-bowel length are related to high mortality rates [8]. Late sequelae, including stenosis, recurrence, and bleeding, are infrequent [18]. Warfarin could be resumed following hematoma regression and improvement in patient’s condition [8]. In our case, the patient recovered well without any postoperative complications and recurrence.

This case underscores the need for clinicians to consider anti-coagulant ileus in the differential diagnosis of patients exhibiting a prolonged INR value, anemia, abdominal pain, and a history of warfarin use, even if the clinical manifestations were atypical. Early recognition and appropriate management could lead to successful patient outcomes.

## Conclusions

We describe a rare case of anti-coagulant ileus, which showed difficulties in making diagnosis due to CT findings and elevated sIL2R levels that suggested a possible malignant lymphoma. This case underlines the importance of considering the possibility of anti-coagulant ileus in patients on long-term anticoagulation medication who exhibit bowel obstruction and elevated PT-INR values.

## Data Availability

Data sharing is not applicable to this article, as no datasets were generated or analyzed.

## Abbreviations

INR: International normalized ratio
Hb: Hemoglobin
CRP: C-reactive protein
PT: Prothrombin time
CT: Computed tomography
sIL-2R: Soluble interleukin 2 receptor
FFP: Fresh frozen plasma
POD: Postoperative day
SISBH: Spontaneous intramural small-bowel hematoma
NOACs: Non-vitamin K oral anticoagulants
US: Ultrasonography
FDG-PET: Fluorodeoxyglucose positron emission tomography
